# Unsupervised Scoliosis Diagnosis via a Joint Recognition Method with Multifeature Descriptors and Centroids Extraction

**DOI:** 10.1155/2018/6213264

**Published:** 2018-09-25

**Authors:** Liyuan Zhang, Jiashi Zhao, Huamin Yang, Zhengang Jiang, Qingliang Li

**Affiliations:** School of Computer Science and Technology, Changchun University of Science and Technology, No. 7089, Weixing Road, Changchun, China

## Abstract

To solve the problem of scoliosis recognition without a labeled dataset, an unsupervised method is proposed by combining the cascade gentle AdaBoost (CGAdaBoost) classifier and distance regularized level set evolution (DRLSE). The main idea of the proposed method is to establish the relationship between individual vertebrae and the whole spine with vertebral centroids. Scoliosis recognition can be transferred into automatic vertebral detection and segmentation processes, which can avoid the manual data-labeling processing. In the CGAdaBoost classifier, diversified vertebrae images and multifeature descriptors are considered to generate more discriminative features, thus improving the vertebral detection accuracy. After that, the detected bounding box represents an appropriate initial contour of DRLSE to make the vertebral segmentation more accurate. It is helpful for the elimination of initialization sensitivity and quick convergence of vertebra boundaries. Meanwhile, vertebral centroids are extracted to connect the whole spine, thereby describing the spinal curvature. Different parts of the spine are determined as abnormal or normal in accordance with medical prior knowledge. The experimental results demonstrate that the proposed method cannot only effectively identify scoliosis with unlabeled spine CT images but also have superiority against other state-of-the-art methods.

## 1. Introduction

Scoliosis is a common spinal abnormality, and it seriously endangers the people's health [1]. Scoliosis recognition is an important premise for preventing the spinal curve from getting worse. Computer-aided diagnosis (CAD) [2] has been a powerful tool to identity scoliosis by analyzing medical imaging. In scoliosis recognition, scoliosis curvature [3] is the most valuable spinal parameter, which can provide the decision value of the normal or abnormal condition. Due to unclear boundaries and degenerative disorders of CT spine images, it is difficult to extract effective scoliosis features from available diagnostic images. A specially designed computer-aided method for accurate scoliosis recognition has a significant research meaningful.

Until recently, many methods have been developed for diagnosing the scoliotic deformity [4–6]. Zhang et al. [7] proposed a semiautomatic scoliosis measurement method to reduce the assessment variability. Hough transform and snake model are integrated together with a shape prior, thus improving the performance and reliability. Little user judgments are still needed. Zukić et al. [8] adopted the Viola–Jones algorithm with candidate filtering to identify scoliosis. Geometric diagnostic features are deduced by detecting vertebral centers. This method extracts the key pathological features, which can further increase robustness of the algorithm, but the optimal parameter largely depends on a manually segmented dataset. Korez et al. [9] presented an automated vertebral detection and segmentation framework with interpolation theory and shape-constrained deformable model. All local optima that correspond to candidate vertebral locations are detected, thereby preserving the vertebral shape. Nonetheless, when severe disorders occur in the CT spine image, the segmentation accuracy will be decreased. Pinheiro et al. [10] proposed a novel computerized methodology with genetic algorithm optimization to evaluate the scoliotic deformity. The ellipse that best fits to the spine curve is introduced. This method can reproduce scoliotic curvatures using the geometric parameters of the underlying ellipses.

To intelligently diagnose scoliosis using machine learning, Glocker et al. [11] developed an approach combining the supervised classification forest and dense probabilistic centroid estimation. Pathological vertebrae images are considered into the discriminative centroid classier. Consequently, the minimum of an estimated centroid location error of 4.4 mm and the detection rate of 86% are obtained. In [12], the authors developed a computer-aided Cobb angle method using deep neural network (DNN) for scoliosis assessment. Enough vertebral patches are needed in the training dataset to automatically determine the slope of the vertebrae. This solution shows a promising result owing to more training data. In [13], a novel BoostNet architecture was designed to estimate vertebral landmarks for adolescent idiopathic scoliosis assessment. Convolutional neural network with statistical theory is used as robust feature extraction. The effectiveness of BoostNet is verified on plenty of spinal X-ray images. In practice, a large amount of high-quality labeled data are crucial to build a better classifier, while the data-labeling processing is very expensive and time-consuming. Additionally, the neighboring vertebrae have similar morphological appearance, which makes them difficult to be distinguished. Therefore, the feature mining of unlabeled CT images is a significant challenge in the scoliosis recognition without manual annotation.

To address this problem, this paper proposes a scoliosis recognition method in the unsupervised setting with unlabeled CT images. The cascade gentle AdaBoost (CGAdaBoost) classifier with multifeature descriptors and distance regularized level set evolution (DRLSE) model are combined into the centroids method. The main contributions of this paper are presented as follows. First, the relationship between individual vertebrae and the whole spine is established using vertebral centroids, which is beneficial to reduce the data-labeling burden on medical staff. Second, three different descriptors are fully combined to achieve more effective features for the CGAdaBoost classifier. Moreover, detected bounding boxes are used as an initial contour of DRLSE to segment vertebral bodies without manual interaction. Our work can provide a feasible and effective scoliosis recognition method for medical intelligence diagnosis.

The remainder of this paper is organized as follows. In Section 2, the gentle AdaBoost classifier and edge-based level set method are briefly introduced.  Section 3 describes the proposed method in detail.  Section 4 focuses on experimental results and discussion, followed by the conclusion and future work in Section 5.

## 2. Related Basic Knowledge

In general, the detection and segmentation of the object are important processes of the recognition task. To achieve a reliable detection result, learning-based technique has been extensively adopted to detect the vertebrae [6, 14–17]. Besides, the vertebral body segmentation exerts a tremendous influence on the extracted centroid result. Level set model is an effective contour evolution method using the image gradient to converge on the object boundary.

### 2.1. Gentle AdaBoost Classifier

Gentle AdaBoost [17] is a type of probability detector with strong robustness. In the aspect of detection accuracy, gentle AdaBoost outperforms discrete and real AdaBoost classifiers owing to a unique weighting update way. Furthermore, a small number of features are only required in the gentle AdaBoost classifier. There is a lower computational complexity. Using the small decision tree as weak classifiers is to improve the generalization ability. The training process of the gentle AdaBoost classifier is described as follows:

*Step 1*. There is a given training dataset *S*={(*x*_1_, *y*_1_),…, (*x*_*n*_, *y*_*n*_)}. *y*_*i*_=0 and *y*_*i*_=1 represent negative and positive samples, respectively.

*Step 2*. Initialize the weight *w*_*i*_ of the training sample, that is, the initial probability distribution of the sample *i*.

(1) $$\begin{array}{c} w_{i}=\left\{\begin{array}{cc} \frac{1}{2m}, & y_{i}=0,\\ \frac{1}{2l}, & y_{i}=1, \end{array}\right. \end{array}$$

where *m* and *l* denote numbers of negative and positive samples, respectively.

*Step 3*. Perform *t*-stage (*t*=1,…, *T*) training on the classifier. *T* is the maximum iteration number. Set *w*_*t*,*i*_ ⟵ *w*_*t*,*i*_/∑_*j*=1_^*n*^ *w*_*t*,*j*_, and renormalize weight such that *w*_*t*_ is a probability distribution. For each feature *j*, the weak classifier *h*_*j*_ is trained according to the weight distribution *w*_*t*_. The minimum error *ε*_*t*_ is got by constructing the error *ε*_*j*_ with weight *w*_*t*_.

(2) $$\begin{array}{c} \varepsilon_{j}=\underset{i}{\sum }w_{i}\;|\;h_{j}\left(x_{i}-{\text{y}}_{i}\right). \end{array}$$

*Step 4*. Update the weight *w*_*t*+1,*i*_ ⟵ *w*_*t*,*i*_ *β*_*t*_^1−*e*_*i*_^ and *β*_*t*_=*ε*_*t*_/(1 − *ε*_*t*_). If *x*_*i*_ is classified correctly, *e*_*i*_ will be equal to zero. Otherwise, *e*_*i*_ is equal to one.

*Step 5*. The final strong classifier is output as follows:

(3) $$\begin{array}{c} h\left(x\right)=\left\{\begin{array}{cc} 1, & \overset{T}{\underset{t=1}{\sum }}\alpha_{t}h_{t}\left(x\right)\geq \frac{1}{2}\overset{T}{\underset{t=1}{\sum }}\alpha_{t},\\ 0, & \text{otherwise,} \end{array}\right. \end{array}$$

where the coefficient *α*_*t*_ is set as *α*_*t*_=log 1/*β*_*t*_.

The weight of each sample is constantly adjusted to form the strongest classifier, thus improving the performance of the classifier and avoiding the overfitting. Hence, the gentle AdaBoost classifier with high efficiency is suitable for the vertebral detection.

### 2.2. Edge-Based Level Set Model

Level set based on edge information [18] constructs an edge detecting function to drive the evolving contour to the desired boundary. Given the initial contour *C*={(*x*, *y*) ∈ *Ω*|*ϕ*(*x*, *y*)=0}, *ϕ* : *Ω*⟶*ℜ* is a level set function (LSF) defined on a domain *Ω*. For the edge distribution of the image, an edge indicator function *g* is defined by

(4) $$\begin{array}{c} g≜\frac{1}{1+{\left|\nabla G_{\sigma }\;*\;I\right|}^{2}}, \end{array}$$

where *I* represents an image. *G*_*σ*_ is a Gaussian kernel with standard deviation *σ*. *∗* denotes a convolution operation to reduce the image noise. The function *g* ∈ [0,1] should take smaller values at object boundaries than other locations. The basic energy function for *ϕ* can be defined as

(5) $$\begin{array}{c} \varepsilon \left(\phi \right)=\text{length}\left(\phi \right)+\text{area}\left(\phi \right), \end{array}$$

where length(*ϕ*) is the length term of the initial contour. Area(*ϕ*) represents the energy of the area inside the contour *C*. Here, both the length term and area term rely on the edge information of the image. These two energy terms can be given as follows:

(6) $$\begin{array}{c} \text{length}\left(\phi =0\right)=\int_{\Omega }g\delta \left(\phi \right)\left|\nabla \phi \right|\;dx,\\ \text{area}\left(\phi \geq 0\right)=\int_{\Omega }gH\left(\phi \right)\;dx, \end{array}$$

where

(7) $$\begin{array}{c} H\left(x\right)=\left\{\begin{array}{cc} 1 & \mathrm{if}\;x>0,\\ 0 & \mathrm{if}\;x<0. \end{array}\right. \end{array}$$

*H*(*x*) is the Heaviside function. And *δ*(*x*) is a Dirac delta smoothing function defined by

(8) $$\begin{array}{c} \delta \left(x\right)=\left\{\begin{array}{cc} \frac{1}{2\varepsilon }\left[1+\cos\;\left(\frac{\pi x}{\varepsilon }\right)\right], & \left|x\right|<\varepsilon ,\\ 0, & \left|x\right|>\varepsilon . \end{array}\right. \end{array}$$

## 3. The Proposed Method

**Figure 1:**
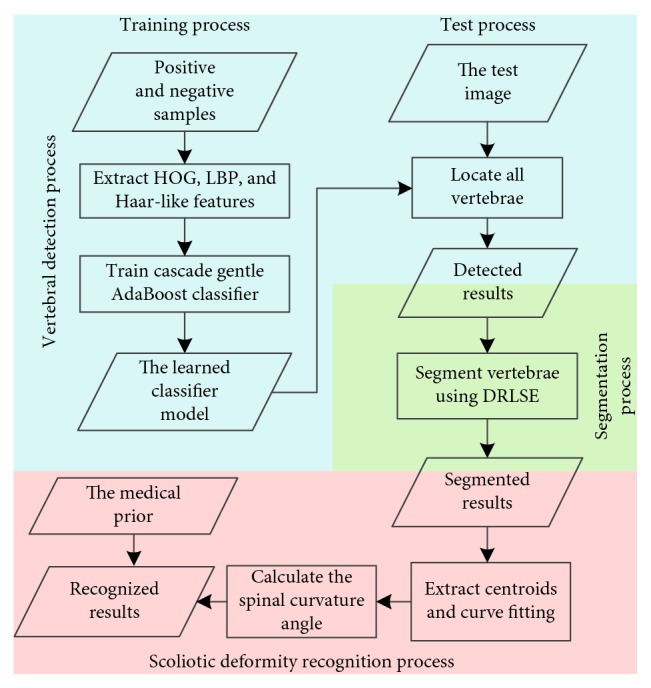
The overview of the CGAdaBoost-DRLSE method.

The motivation of this paper is to accurately recognize scoliosis from unlabeled CT images. For convenience, the proposed method is called as CGAdaBoost-DRLSE.  Figure 1 depicts an overview of the CGAdaBoost-DRLSE method. The proposed method is comprised of three main stages. The first stage is the automated vertebrae detection, including training and testing processes. Diversified training samples and multifeature descriptors are considered to train the CGAdaBoost classifier and detect all vertebral bodies in the CT spine image. The second stage is the vertebral region segmentation. DRLSE strongly depends on a relatively accurate initialization. Consequently, the detected bounding boxes are used as the initial contours of DRLSE. Acquired segmentation results further serve for vertebral centroids extraction. Vertebral centroids are extracted to connect the whole spine, which can make scoliotic deformity recognition more accurate. After spinal curve fitting, calculating the angle between two tangents is to measure the spinal curvature angle, thereby diagnosing spinal abnormalities.

### 3.1. Vertebrae Detection

**Figure 2:**
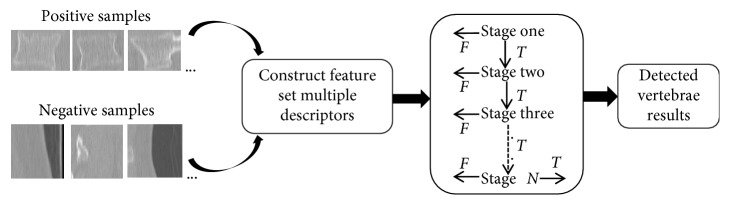
Schematic of the cascade gentle AdaBoost detector.

We make the first attempt to use the cascade gentle AdaBoost detector with multifeature fusion to detect vertebrae. The classifier with the cascade structure is essentially a degenerated decision tree, which arranges a series of strong AdaBoost classifiers from simple to complex [19]. By continuously training, each strong classifier will have a higher detection rate and lower false-positive rate.  Figure 2 illustrates the basic schematic of a cascade classifier.

In the training process, only positive samples of the previous classifier will be transmitted into the next classifier to continue learning. Then, some subwindows belonging to positive samples in each classifier are output as the detected vertebrae. On the contrary, subwindows belonging to negative samples will be rejected directly. Obviously, the cascade classifier can overcome the problem of the imbalanced sample and significantly improve the efficiency of the detector. The training process of the CGAdaBoost classifier is briefly described, as shown in Algorithm 1.

### 3.2. Multifeature Fusion

Currently, Haar-like [20], local binary pattern (LBP) [21], and histogram of oriented gradients (HOG) [22] descriptors have become the most useful digital image feature methods in object recognition. Among them, Haar-like descriptor is used to describe the image intensity differences. LBP is suitable for describing the local texture feature of the image. HOG descriptor can better describe appearance and shape of the object, that is, the local gradient or the distribution of the edge direction.

Although each single descriptor is highly efficient, the extracted features are difficult to accurately distinguish vertebral and nonvertebral regions from spine CT images with low contrast. Therefore, to make full use of the advantage of each feature descriptor, we present a multifeature fusion way. HOG, LBP, and Haar-like features will be combined together to construct a feature vector, thus generating the optimal feature set. Before fusing, these features will be normalized for the facility of computation. The final feature set *F*_final_ is formally expressed as

(9) $$\begin{array}{c} F_{\text{final}}=F_{\text{Haar}}+F_{\text{LBP}}+F_{\text{HOG}}. \end{array}$$

Haar-like adopts the black-and-white feature template to perform sliding detection on the image. The integral graph is employed to realize the fast summation of subregions. And the sum of the pixel in the white region subtracts the sum of the pixel in the black region as feature value *F*_Haar_, which can be described as

(10) $$\begin{array}{c} F_{\text{Haar}}=\text{sum}\left(R_{\text{white}}\right)-\text{sum}\left(R_{\text{black}}\right). \end{array}$$

For the LBP descriptor, the image is divided into several subregions. Then, the LBP feature of each pixel in the subregion is extracted. The statistical histogram of each subregion constitutes the texture feature vector of the whole image. A mathematical description of the LBP can be given as follows:

(11) $$\begin{array}{c} F_{\text{LBP}}=\overset{P-1}{\underset{p=0}{\sum }}2^{p}s\left(f_{\text{p}}-f_{\text{c}}\right)=\left\{\begin{array}{cc} \overset{P-1}{\underset{p=0}{\sum }}2^{p}, & \mathrm{if}\;\;f_{\text{p}}\geq f_{\text{c}},\\ 0, & \text{else,} \end{array}\right. \end{array}$$

where *p* is the *p*th pixel of the subregion. *f*_p_ and *f*_c_ represent the gray value of the neighboring pixel and central pixel, respectively.

HOG descriptor also divides the image into several small connected regions. The gradient direction (or edge direction) histogram of the pixels in each region is calculated, thereby combining these histograms as a feature vector. The gradient vector of the HOG descriptor can be obtained as

(12) $$\begin{array}{c} F_{\text{HOG}}=\nabla f\left(x,y\right)=\left[\begin{array}{c} \frac{\partial f\left(x,y\right)}{\partial x}\\ \frac{\partial f\left(x,y\right)}{\partial y} \end{array}\right]=\left[\begin{array}{c} f_{x}\\ f_{y} \end{array}\right], \end{array}$$

where *f*(*x*, *y*) denotes a positive sample; *f*_*x*_ and *f*_*y*_ represent the gradient vectors of *x* and *y* directions, respectively.

### 3.3. Vertebral Body Segmentation

Vertebrae segmentation is vitally important to extract centroids for scoliosis recognition. DRLSE [23] is an edge-based level set evolution method. A distance regularization term is introduced into the energy function as a penalty term. DRLSE can produce a better effect of edge detection and avoid the reinitialization process. Aiming at the low contrast and high noise of CT image, it is necessary to employ a contrast limited adaptive histogram equalization (CLAHE) method [24] to enhance the vertebral region before segmenting.

To further reduce manual intervention, detected bounding boxes are viewed as the initial contour *C* of DRLSE. The energy function is minimized by solving the following gradient flow equation:

(13) $$\begin{array}{c} \frac{\partial \phi }{\partial t}=\mu \mathrm{div}\left(d_{p}\left(\left|\nabla \phi \right|\right)\nabla \phi \right)+\lambda \delta_{\varepsilon }\left(\phi \right)\mathrm{div}\left(g\frac{\nabla \phi }{\left|\nabla \phi \right|}\right)+\alpha g\delta_{\varepsilon }\left(\phi \right), \end{array}$$

where the first term is the distance regularization term, and *μ* > 0 is the coefficient of the distance regularization term; the second term stands for the gradient flow of the length term, and *λ* denotes the weighted coefficient of the length term; the third term is the gradient flow of the area term, and *α* is the weighted coefficient of the area term; and the div(·) is the divergence operator.

In (13), the definition of *d*_*p*_ is given by

(14) $$\begin{array}{c} d_{p}\left(s\right)=\frac{p^{\prime }\left(s\right)}{s}, \end{array}$$

where the potential function *p*(·) should have minimum points at *s* = 0 and *s* = 1. The flow in (13) has a diffusion effect on the level set function *ϕ*. This diffusion is termed as forward-and-backward diffusion, which adaptively increases or decreases ∇*ϕ* to maintain the desired shape of the function *ϕ*, thus avoiding the effect of bad edges.

A preferable potential function can keep the sign distance function smoothing in the distance regularization term, which can be expressed as

(15) $$\begin{array}{c} p\left(s\right)=\left\{\begin{array}{cc} \frac{1}{2\pi^{2}}\left(1-\cos\left(2\pi s\right)\right), & \mathrm{if}\;\;s\leq 1,\\ \frac{1}{2}{\left(s-1\right)}^{2}, & \mathrm{if}\;\;s\geq 1. \end{array}\right. \end{array}$$

As a consequent, the vertebral body region is obtained by evolving iteratively. It is noticed that the combination of detection and segmentation solves not only the sensitivity of initialization, but also quickly and accurately converges on vertebral boundaries.

### 3.4. Scoliosis Recognition with the Medical Prior Knowledge

To establish the relationship between the individual vertebrae and the whole spine, we extract vertebral centroids as a shared feature from the segmented result. This work highlights the importance of the vertebral centroid. Let *I*(*x*, *y*) be a segmented binary mask, where its size is *m* × *n*. The centroid coordinate (*k*, *l*) of each vertebral body can be expressed as

(16) $$\begin{array}{c} k=\frac{\sum_{x}^{m}\sum_{y}^{n}xI\left(x,y\right)}{\sum_{x}^{m}\sum_{y}^{n}I\left(x,y\right)},\\ l=\frac{\sum_{x}^{m}\sum_{y}^{n}yI\left(x,y\right)}{\sum_{x}^{m}\sum_{y}^{n}I\left(x,y\right)}, \end{array}$$

where $I\left(x,y\right)=\left\{\begin{array}{c} 1,\;\left(x,y\right)\in \text{object},\\ 0,\;\left(x,y\right)\in \text{background}. \end{array}\right.$ All vertebral centroids are computed by solving (16) iteratively.

**Figure 3:**
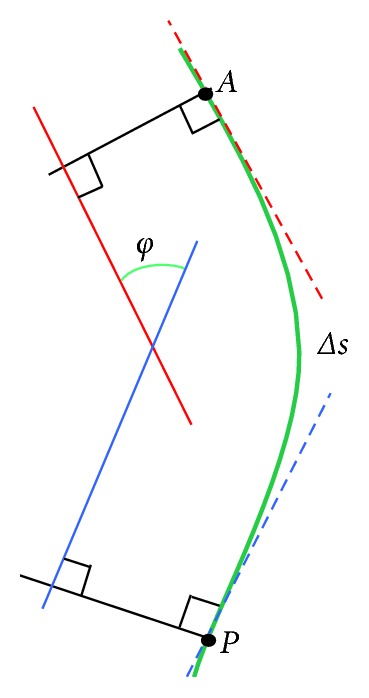
The schematic diagram of the curvature angle.

After extracting vertebral centroids, the least squares method [25] is adopted to construct the spinal curve. In Figure 3, suppose that the spinal curve *y*  =  *f*(*x*) has a continuous derivative. And the points *A* and *P* represent anterior and posterior vertebral centroids, respectively. Δ*s* denotes the length of arc *AP*, and *φ* is the angle between two tangents of the curve. Therefore, the curvature of *y* at the point (*x*, y) is given by

(17) $$\begin{array}{c} k=\left|\frac{d\varphi }{ds}\right|=\frac{\left|y^{\prime \prime }\right|}{{\left(1+{y^{\prime }}^{2}\right)}^{3/2}}, \end{array}$$

where *dφ*=(*y* ^″^/1+*y*′^2^) *dx*. In centroid measurement method, the spinal curvature angle is defined as the angle between two tangents at centroid points of two terminal vertebrae, which can be calculated as follows:

(18) $$\begin{array}{c} \varphi =\int_{a}^{b}\frac{y^{\prime \prime }}{1+{y^{\prime }}^{2}}\;dx. \end{array}$$

According to the prior medical treatment, viewed from the coronal, the spinal curve looks like a straight line. Generally, if the curvature angle is greater than ten degrees, the spine will be diagnosed as scoliosis:

(19) $$\begin{array}{c} \mathrm{diagnosis}\;\;\mathrm{result}\;\;1=\left\{\begin{array}{cc} \mathrm{scoliosis,} & \mathrm{if}\;\;\varphi \geq 10^{{}^{\circ}},\\ \mathrm{normal}, & \mathrm{if}\;\;\varphi <10^{{}^{\circ}}. \end{array}\right. \end{array}$$

In addition, viewed from the sagittal, the curve of the spine is in “S” shape. There are three normal curvatures of spine, including cervical lordosis (35° to 45°), thoracic kyphosis (20° to 45°), and lumbar lordosis (40° to 60°) [26]:

(20) $$\begin{array}{c} \mathrm{diagnosis}\;\;\mathrm{result}\;2=\left\{\begin{array}{cc} \mathrm{cervical}\;\mathrm{lordosis,} & \mathrm{if}\;\;\varphi \notin \left[35^{{}^{\circ}},45^{{}^{\circ}}\right],\\ \mathrm{thoracic}\;\;\mathrm{kyphosis,} & \mathrm{if}\;\;\varphi \notin \left[20^{{}^{\circ}},45^{{}^{\circ}}\right],\\ \mathrm{lumbar}\;\;\mathrm{lordosis,} & \mathrm{if}\;\;\varphi \notin \left[40^{{}^{\circ}},60^{{}^{\circ}}\right] \end{array}\right. \end{array}$$

The measurement from the coronal view is focused on the diagnosis of scoliosis, while the measurement from the sagittal view refers to the diagnosis of lumbar lordosis, thoracic kyphosis, and cervical lordosis.

## 4. Experimental Results and Discussion

### 4.1. Data Description and Experimental Platform

To verify the effectiveness and feasibility of the proposed method, a variety of experiments are conducted on about 500 spine CT images to automatically recognize scoliotic deformity. These images are from 20 subjects (11 males and 9 females; age range 18–56 years) of available spine CT volumes on the publicity platform *SpineWeb* [27]. The volume size is 512 × 512 × (100 – 240). The view of the spine image is limited to 5–20 vertebrae. Each dataset may include high-grade scoliosis, kyphosis, and fractures, which is along with ground-truth centroid of each vertebra. All experiments are implemented in the Matlab 2014a platform, which run on Microsoft Windows 7 64-bit operating system with 3.20 GHz Intel® Xeon® CPU, 8 Gbyte RAM.

### 4.2. The Analysis of Experimental Results

**Figure 4:**
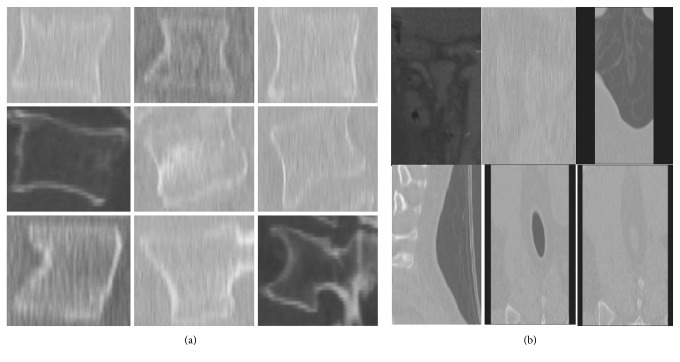
Parts of training samples: (a) positive samples; (b) negative samples.

In the vertebral detection experiment, we only select the vertebral bodies as positive samples without considering the spinal cord, ribs, and sacrum. The positive and negative samples are created by the Training Image Labeler Toolkit of Matlab 2014a, resulting in 520 positive samples and 1058 negative samples.  Figure 4 shows a part of positive and negative samples.

The positive samples contain various parts (cervical, thoracic, and lumbar) of the whole spine. Additionally, vertebrae images with different views (sagittal and coronal), arbitrary contrasts, and lesions are also considered as positive samples. We select nonvertebral regions from CT images as negative samples. As far as possible to increase the distinguishability of interclass samples, diverse features will be provided for classifying.

**Figure 5:**
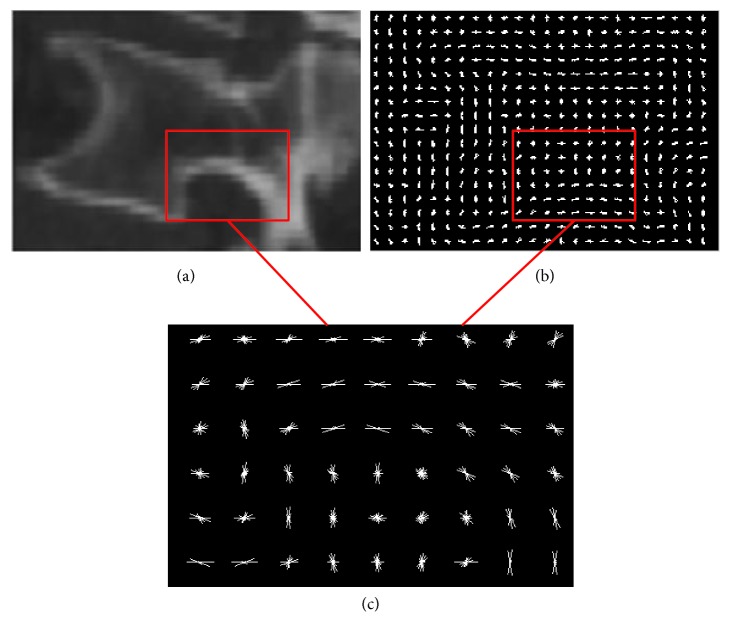
The result of the HOG descriptor: (a) CT vertebral image; (b) the visualization of HOG; (c) local amplification result.

**Figure 6:**
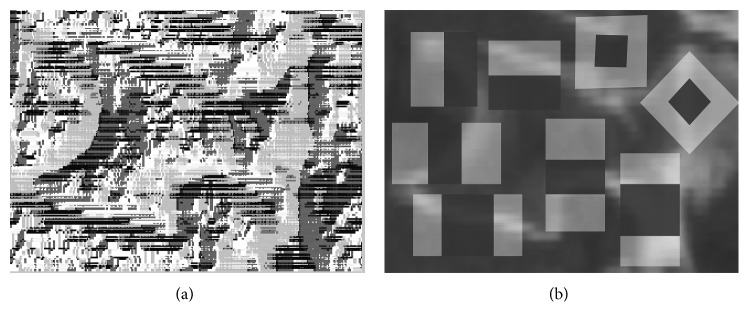
Results of LBP and Haar-like descriptors: (a) the visualization of LBP; (b) Haar-like feature with edge features, center-surround features, and line features.

For feature extraction, Figures 5 and 6 show extracted results of HOG, LBP, and Haar-like from the same positive sample. Here, the 8 × 8 cell constitutes a HOG block. From Figure 5, it can be seen that the HOG feature better describes the edge gradient and shape information of the vertebrae.

Figure 6(a) illustrates the texture image via LBP. The vertebral edge can be visualized clearly. However, Haar-like is an effective method to reflect the change of image gray. In Figure 6(b), two edge features, two center-surround features, and four line features are extracted as the final Haar-like feature. Furthermore, we have taken into account the complete feature set for the classifier as only combining HOG, LBP, and Haar-like descriptors. To do this, the intensity, appearance, and shape of the individual vertebrae can be fully expressed.

**Figure 7:**
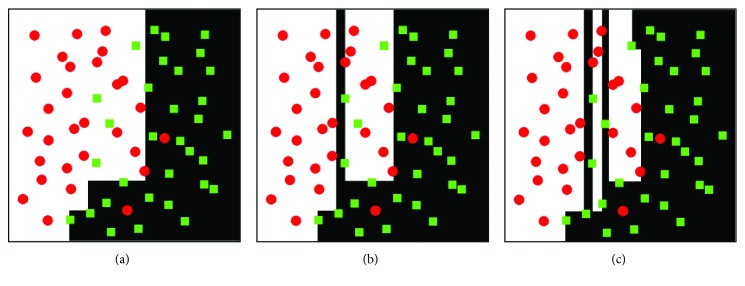
The training process of the optimal classifier: (a) the weak classifier with false-positive rate 0.1; (b) the weak classifier with false-positive rate 0.08; (c) the strong classifier with false-positive rate 0.03.

In the training process, the gentle AdaBoost classifier built a powerful classifier with high accuracy through several simple weak classifiers.  Figure 7 shows the processes of training weak classifiers and the optimal classifier. Red dots and green boxes represent two classes. After numerous iterations, red dots and green boxes are classified into two regions (white region and black region). Figures 7(a) and 7(b) display weak classifiers with different false-positive rates. Actually, the construction of the optimal classifier is to find the appropriate classifier parameter. Hence, the classifier has the lowest false-positive rate of 0.03 for all samples, which is as shown in Figure 7(c).

The CGAdaBoost classifier captured the shape and pathological features of the vertebrae. At the same time, the vertebrae of sagittal and coronal views also are detected from CT spine images. We obtain the optimal parameter of the classifier after several experiments. To reduce the loss of the vertebrae, the true-positive rate should be set to a larger value. Likewise, the smaller the value of the false-positive rate is, the less the number of the falsely detected vertebrae is. As a result, TruePositiveRate (true-positive rate) is set to 0.9, and FalseAlarmRate (false-positive rate) is set to 0.03. The number of the training stage (NumCascadeStages) is set to 10 according to the total number of samples. In our implementation, the subwindow size is experimentally set to 90 × 80. Only in this way, the initial contour of the DRLSE method is closer to the edge of the vertebrae such that the final segmentation results are more accurate to serve for vertebral centroids extraction.

**Figure 8:**
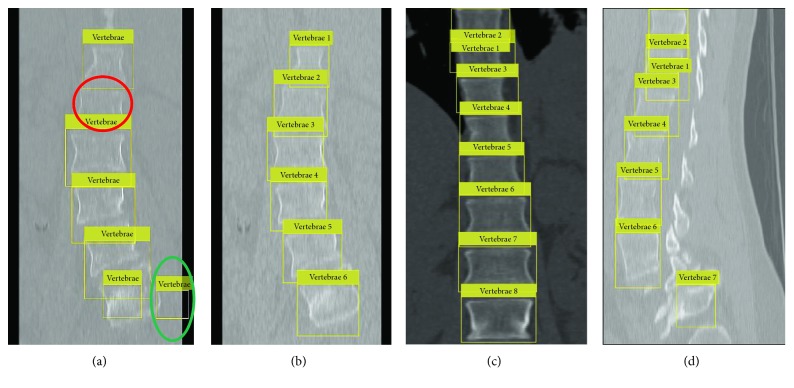
Detection results of the CGAdaBoost classifier with FalseAlarmRate 0.03 and TruePositiveRate 0.9: (a) the detection result with single feature on the coronal view (a red circle for the undetected vertebrae and a green circle for the false detected vertebrae); (b) the detection result with multifeature on the coronal view; (c) the detection result with multifeature on the high-contrast coronal view; (d) the detection result with multifeature on the sagittal view.

**Figure 9:**
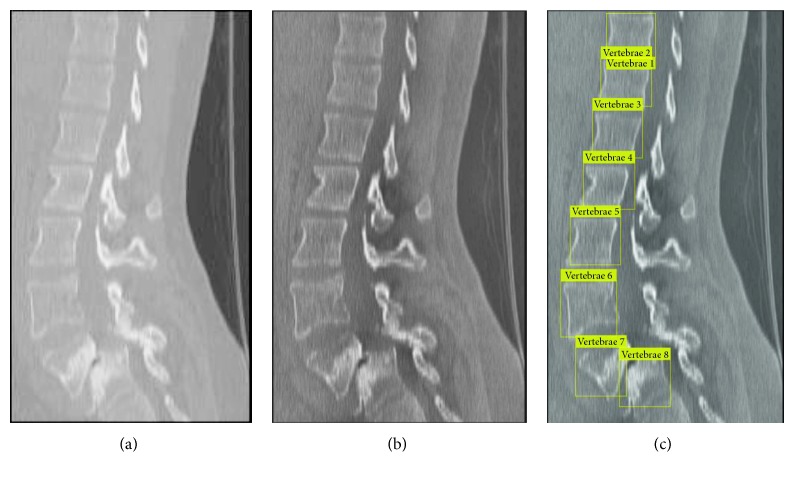
Another detected result: (a) original CT image; (b) the enhanced image by the CLAHE method; (c) the final detected result.

Various detection results with different feature descriptors on sagittal and coronal planes are illustrated in Figure 8. When the single HOG feature is used to detect the vertebrae on the coronal view, there are undetected vertebrae (red circle) and false detection vertebrae (green circle) in Figure 8(a). In contrast, Figure 8(b) shows better the detected result with multifeature fusion. To demonstrate the diversity of our result, the detected result with multifeature on the high-contrast coronal image is shown in Figure 8(c). It can be seen from Figure 8(d) that in spite of vertebrae 7 having a complex shape deformation, it can still be detected.  Figure 9 shows the detected result after enhancing the image of CLAHE. It is evident that the enhanced CT image can make detection more accurate and provide suitable initial contours for the subsequent DRLSE model. It is worth noting that the scanning order of the testing process is from left to right and from top to bottom on the test image. Then, the scanned subwindow belonging to the vertebrae will be labeled using numbers. The aim of numbering is to facilitate different parts extraction from the whole spine.

**Figure 10:**
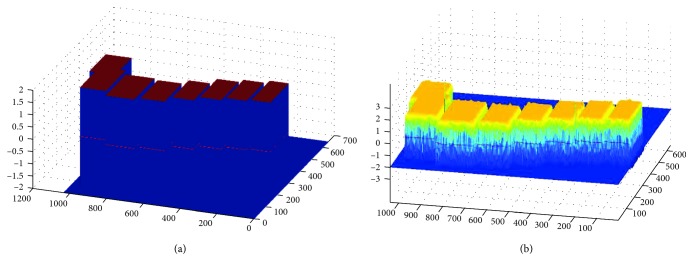
The evolution result of DRLSE: (a) the initial level set function; (b) the final level set function.

We applied the DRLSE method to segment the vertebrae from spine CT images without any user intervention. On the basis of vertebral detected results, located bounding boxes are regarded as the initial contour *C* to eliminate the sensibility of initialization of DRLSE. In the segmentation experiment, we set the appropriate coefficient *μ*=0.04 of the distance regularization term. The weighted coefficients of the length term *λ* and area term *α* are set to 5 and 1.5, respectively. Scale parameter *ε* in Gaussian kernel is set to 1.5. To visualize the curve evolution process, Figure 10 displays the evolution results of the initial and the final level set functions. The red curve represents a zero level set function.

**Figure 11:**
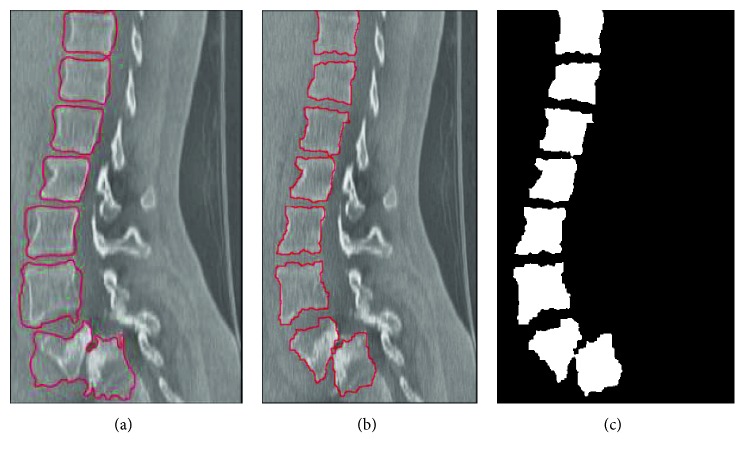
The segmentation result on the sagittal view: (a) the contour with 100 iterations; (b) the final contour with 200 iterations; (c) the final segmentation result.

**Figure 12:**
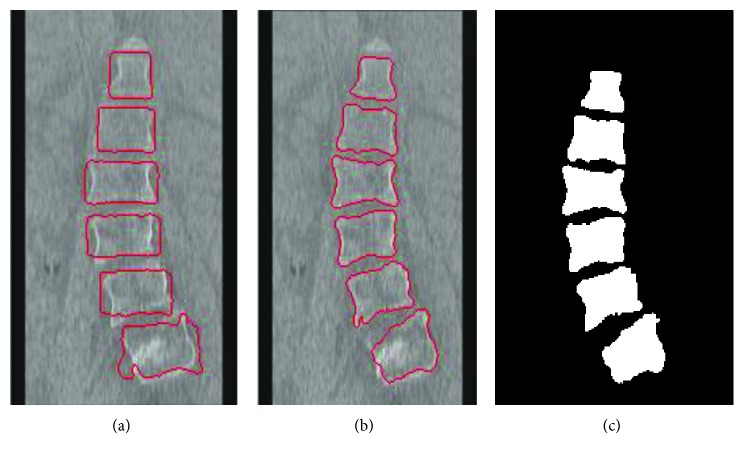
The segmentation result on the coronal view: (a) the contour with 100 iterations; (b) the final contour with 200 iterations; (c) the final binary segmentation result.

By adjusting appropriate parameters, Figures 11 and 12 show two spinal segmentation results.  Figure 11(a) presents the evolutionary result of 100 iterations on the sagittal view.  Figure 11(b) shows the evolution result of 200 iterations.  Figure 11(c) shows the binary segmentation result after 200 iterations.  Figure 12 displays the segmented result on the coronal view of the spine CT image. Obviously, it can be observed that the initial contour can quickly converge on vertebra boundaries to improve the segmentation accuracy. In addition, less iteration is needed to obtain the final converged result, thereby avoiding a larger displacement of the initial contour.

**Figure 13:**
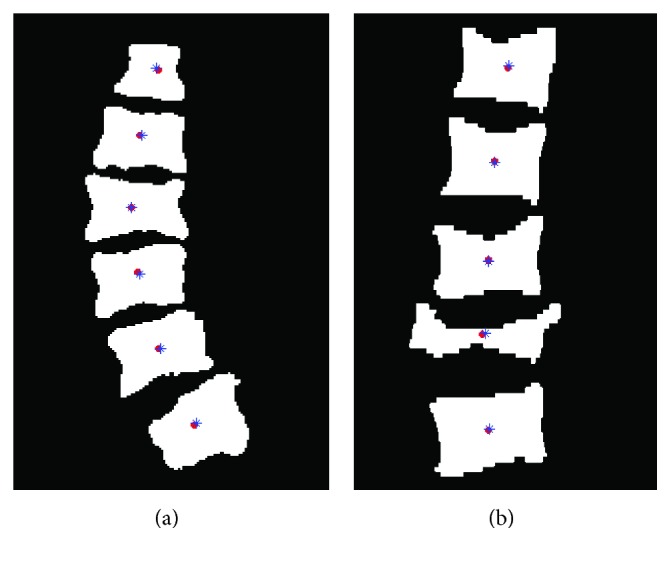
Comparison results of the extracted centroids (red solid point) with the ground-truth centroids (blue star): (a) the result without the vertebral pathology case; (b) the result with the severe vertebral fracture case.

The final goal of our method is to represent the detailed shape of the spinal curve using the centroid method. It should be pointed out that we would select the appropriate CT slice of the spine image in order to extract vertebral centroids.  Figure 13 shows a comparison result between the extracted centroid and ground-truth centroid. It can be seen that all extracted centroids of CGAdaBoost-DRLSE with multifeature fusion are nearly consistent with the ground truth. Only less extracted centroid has a larger error because of the severe vertebral fracture, which is seen in Figure 13(b).

**Figure 14:**
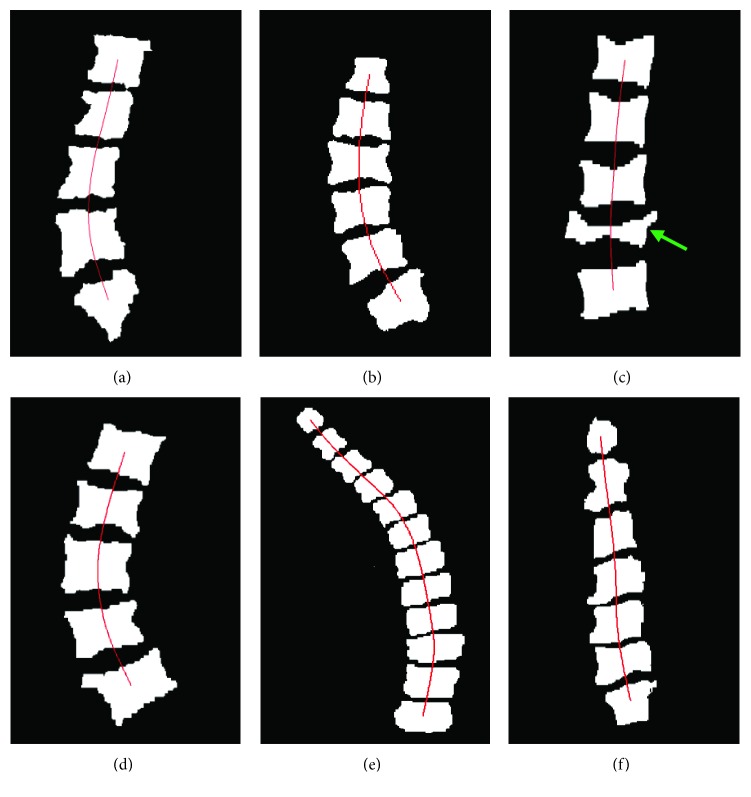
Curve fitting results of various parts in the whole spine: (a) sagittal lumbar curvature; (b)–(d) coronal lumbar curvature; (e) coronal thoracic curvature; (f) coronal cervical curvature.

Furthermore, we extract the lumbar vertebrae, cervical vertebrae, and thoracic vertebrae from the whole spine. The corresponding spinal curve is calculated by the least squares method.  Figure 14 displays the curve fitting results of various parts of the whole spine. The curve of the lumbar (i.e., from L1 to L5) on the sagittal view is presented in Figure 14. The curve of the lumbar (i.e., from T12 to L5) on the coronal view is given in Figure 14. Figures 14 and 14 show the curves of the lumbar on the coronal view. The severe scoliosis curve of the thoracic (i.e., from T1 to T12) on the coronal view is shown in Figure 14. The curve of the cervical (i.e., from C1 to C7) on the coronal view is shown in Figure 14. Specially, from Figure 14, we can see that, even if there are some distorted vertebrae (indicated by the arrow), it has no influence on the whole spinal curvature.

After curve fitting, the angle between two tangents to the curve is determined as the spinal curvature angle.  Table 1 lists a part of spinal abnormalities diagnostic results in Figure 13. Comparing the spinal curvature angle with the prior medical treatment, the spine is decided as normal or abnormal. By analyzing the above results, our method shows promising scoliosis recognition results and also can be applicable to other image patterns. Meanwhile, this method also diagnoses lordosis and kyphosis by observing the spinal curvature from a sagittal view. The resulting curvature of the spine is not affected by bone lesions, without the need to manually identify the endplate vertebrae.

### 4.3. The Assessment of Performance

**Figure 15:**
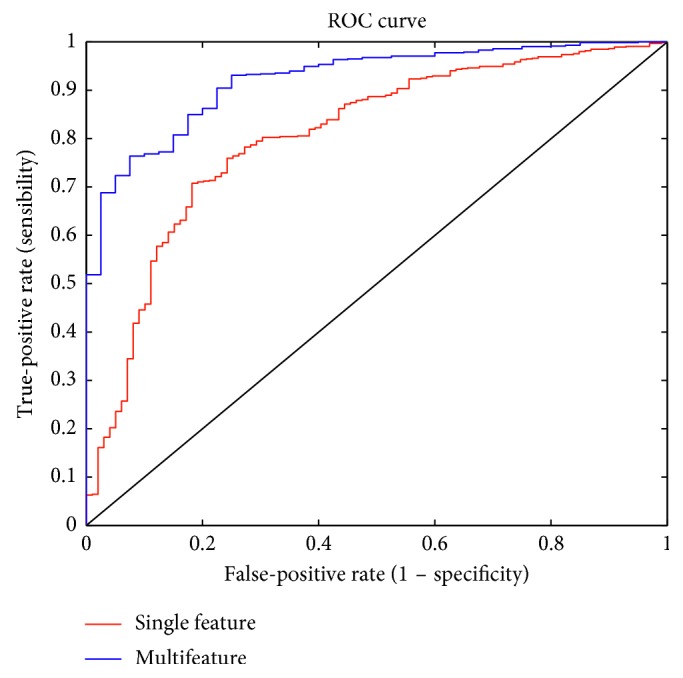
The ROC curve on different methods.

To evaluate the performance of the cascade gentle AdaBoost classifier, the receiver operator characteristic (ROC) is used as an evaluation criterion. ROC intuitively shows the compromise between true-positive rate and false-positive rate for the classification model.  Figure 15 compares the ROC curve of CGAdaBoost with a single-feature and multifeature fusion. It is obvious that the ROC of the multifeature fusion method is higher than a single-feature method. This further reveals that the detected vertebrae are more accurate by the CGAdaBoost classifier with multifeature fusion.

**Figure 16:**
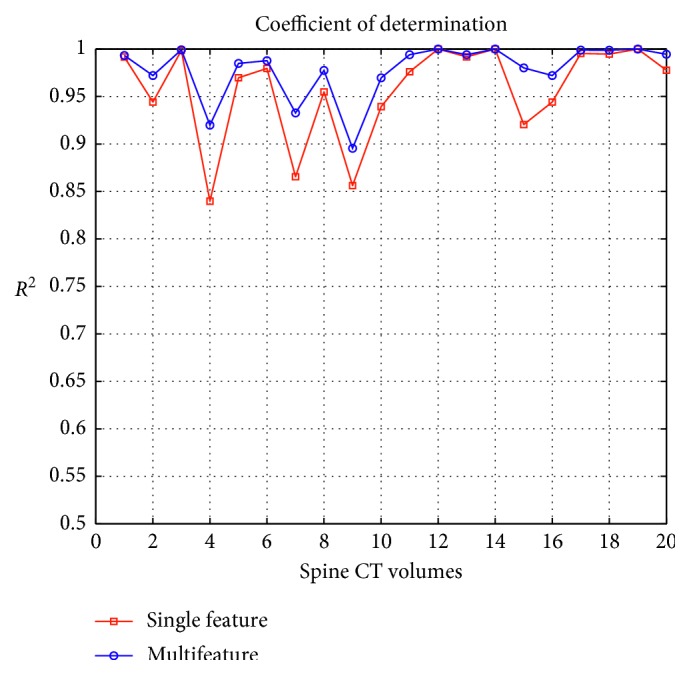
*R* ^2^ of different methods on twenty subjects.

**Figure 17:**
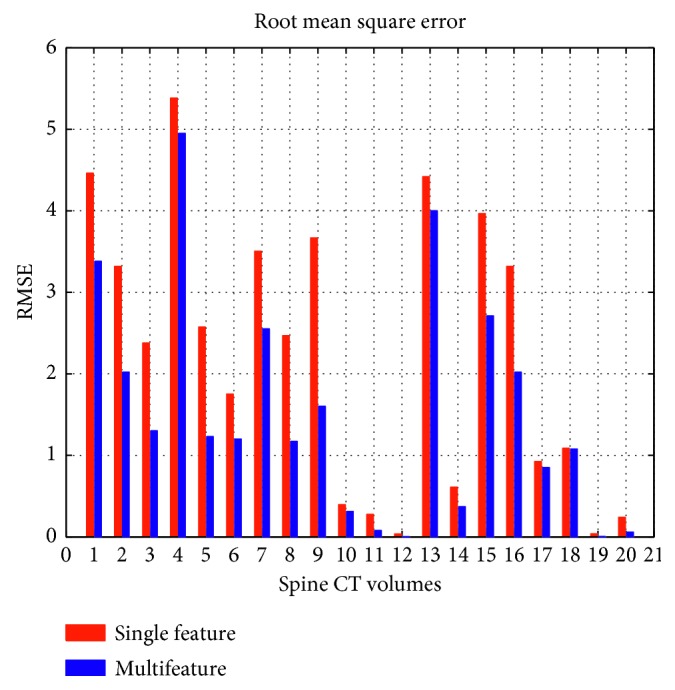
RMSE of different methods on twenty subjects.

Additionally, to verify the credibility of the spinal curve fitting, using two evaluation criteria is to comprehensively assess the quality of curve fitting. One is the coefficient of determination (*R* ^2^). It is a statistic of goodness of fit. The value range is from 0 to 1. And the larger the value is, the better the fitting effect is. The other is root mean squared error (RMSE) that is the relative deviation between the predicted value and the real value. The mathematical expressions of two criteria are given as follows:

(21) $$\begin{array}{c} R^{2}=1-\frac{\sum_{\text{i}=1}^{n}{\left(v_{i}^{\prime }-v_{i}\right)}^{2}}{\sum_{\text{i}=1}^{n}{\left(v_{i}-\bar{v}\right)}^{2}},\\ \text{RMSE}=\sqrt{\frac{1}{n}\overset{n}{\underset{\text{i}=1}{\sum }}{\left(v_{i}-v_{i}^{\prime }\right)}^{2}}. \end{array}$$

where *v* and *v*′ indicate the real value and the fitting value, respectively, and $\bar{v}$ is the mean value of *n* samples. By calculating the values of *R* ^2^ and RMSE, the results of different methods on twenty spine CT volumes are displayed in Figures 16 and 17, respectively.

In Figure 16, we can see that no matter which method, it gains relatively higher *R* ^2^ on each volume. The *R* ^2^ of multifeature fusion is closer to one. It reflects that the spine curve fitting has better goodness of fit. Meanwhile, from Figure 17, we clearly know that multifeature fusion outperforms a single-feature method in terms of RMSE on each dataset. It is because that better spine curve fitting may depend on accurate vertebral centroids. This also confirms the effectiveness and quality of curve fitting in the proposed method.

Furthermore, the detection accuracy rate and centroid location error are employed to further assess the proposed method. From the total 231 vertebrae, CGAdaBoost-DRLSE with single feature detects 227 vertebrae, resulting in the detection accuracy rate of 98%. By contrast, the multifeature fusion successfully detects 229 vertebrae and has the detection accuracy rate of about 99%. The centroid location error is computed using the Euclidean distance between extracted centroids and ground-truth centroids. The single-feature method achieves an average centroid location error of 1.51 mm. The multifeature fusion method has the mean centroid location error of 0.87 mm. Two related methods reported by Korez et al. [9, 11] use the same database as our method for fair comparison. The performance comparison result is summarized in Table 2.

In Table 2, the proposed method is superior to other competitive methods in terms of the detection accuracy rate. This is because that vertebral shape variations and pathological features are fully fused. Thus, effective vertebrae features are trained to improve the detection accuracy. On account of inadequate estimation using the dense probabilistic centroid estimation method in [11], our method potentially produces less centroid location error. By compared with interpolation theory and shape-constrained method [9], we found that CGAdaBoost-DRLSE with multifeature is slightly superior to the result of [9], which is most probably due to incorrect segmentation of some smaller vertebrae. Overall, the proposed method can automatically detect and segment vertebrae from CT images. In particular, for a relatively small number of subjects with some pathological vertebra, it can provide accurate spinal curvature to recognize scoliosis deformity from unlabeled CT images. Moreover, any manual landmark is not required. These unknown CT images can be labeled as abnormal or normal through this unsupervised method.

## 5. Conclusion

This paper proposes an unsupervised scoliosis recognition method with unlabeled CT images to improve the accuracy. The CGAdaBoost-DRLSE method consists of vertebral bodies' detection, segmentation, and centroids extraction. Firstly, diversified training samples and multifeature descriptors are considered to achieve better detection results in the cascade gentle AdaBoost classifier. Then, located bounding boxes represent the initial contour of DRLSE to eliminate the sensitivity of initialization and quickly converge on vertebral boundaries. Finally, vertebral centroid extraction and curve fitting are performed to compute the spinal curvature angle, thereby recognizing scoliosis with the prior medical treatment. Experimental results have demonstrated that the proposed method can effectively and accurately diagnose scoliosis deformity and reduce the need for manual landmark. Besides, the proposed method also is suitable for clinical work with acceptable results and serves as a quick guideline for nonexperts. In future work, we will extend the proposed method to the three-dimensional case by introducing spatial information of CT spine volume and classify various vertebral fractures by the designed multiclass classifier.

## Figures and Tables

**Algorithm 1:**
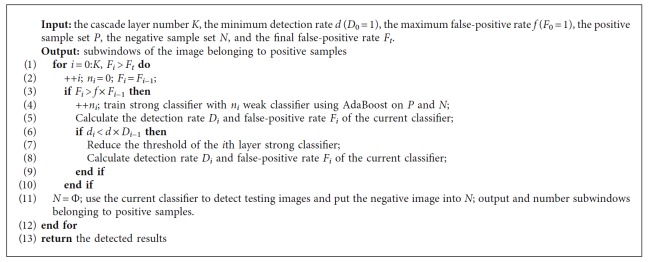
The training process of the cascade gentle AdaBoost classifier.

**Table 1:** A part of diagnosis results.

Binary mask	The curvature angle	The diagnosis result
Figure 14	Sagittal lumbar 40.4°	Normal
Figure 14	Coronal lumbar 30.5°	Abnormal
Figure 14	Coronal lumbar 3.2°	Normal
Figure 14	Sagittal lumbar 44.6°	Normal
Figure 14	Coronal thoracic 55.2°	Abnormal
Figure 14	Coronal cervical 2.6°	Normal

**Table 2:** The performance comparison of our methods and other related works.

Methods				
Evaluation criteria	Supervised classification forests [11]	Interpolation theory + shape-constrained [9]	Single-feature + CGAdaBoost-DRLSE	Multifeature + CGAdaBoost-DRLSE
Detection accuracy rate	86%	97%	98%	99%
Centroid location error	4.4 mm	1.1 mm	1.51 mm	0.87 mm

## Data Availability

The experimental datasets analysed during this study are available in the publicity platform SpineWeb, (http://spineweb.digitalimaginggroup.ca/dataset.html).
